# Differences in Health-Related Quality of Life and Physical Condition of Two Community-Based Exercise Programs in Subjects with Cardiovascular Risk Factors: A Prospective Observational Cohort Study

**DOI:** 10.3390/jpm12111894

**Published:** 2022-11-12

**Authors:** Esther García-Sánchez, Vicente Ávila-Gandía, F. Javier López-Román, Jacobo Á. Rubio-Arias, Juan F. Menarguez-Puche

**Affiliations:** 1 Fundación para la Formación e Investigación Sanitaria de la Región de Murcia (FFIS), 30005 Murcia, Spain; 2 Sports Physiology Department, Catholic University of Murcia (UCAM), 30107 Murcia, Spain; 3 Biomedical Research Institute of Murcia (IMIB-Arrixaca), 30005 Murcia, Spain; 4 Health Research Centre, Department of Education, Faculty of Educational Sciences, University of Almería, 04120 Almería, Spain; 5 Primary Health Care Center “Profesor Jesús Marín”, Molina de Segura, 30500 Murcia, Spain

**Keywords:** physical activity, health-related quality of life, physical condition, cardiovascular risk

## Abstract

We compared the effect of two community-based physical activity (PA) programs on health-related quality of life (HRQL) and physical condition in people with cardiovascular risk factors. Fifty-one subjects participated in the “ACTIVA Murcia” AM_3_ program characterized by non-individualized training loads for 3 months, and forty-two participated in the AM_6_ program characterized by individualized progressive training loads for 6 months. Both programs included a 6-month follow-up period without PA. HRQL was assessed with the Short Form 36 Health Survey (SF-36) and physical condition by VO_2_ max, strength, flexibility, and balance. Participants in the AM_6_ program as compared with those in the AM_3_ program showed significantly higher scores in the subscales of physical functioning, mental health, energy/vitality, and general health. Mental health and general health at 6 months of follow-up were also scored significantly higher by AM_6_ participants. VO_2_ max and flexibility improved more in the AM_6_ group, whereas strength was better in the AM_3_ group. Half of the participants in the AM_6_ program expressed a strong willingness to continue exercising vs. 38% in the AM_3_ program. In this study, a community-based PA program with individualized progressive training loads of 6-month duration showed a more favorable impact on HRQL than a 3-month non-individualized PA program.

## 1. Introduction

Health-related quality of life (HRQL) is a key benefit of physical activity (PA). Much research including recent systematic reviews and meta-analyses provide strong evidence of a consistent positive association between PA and HRQL [1,2,3,4]. Sedentary behavior and physical inactivity are among the leading modifiable risk factors for cardiovascular disease, all-cause mortality, and cardiovascular mortality independently from age, sex, and pre-existing cardiovascular disorders [5,6]. Limited physical activity, especially the inability to take regular walks out of one’s home, is also associated with a number of unfavorable metabolic effects that increase the risk of disabling and severe disorders, such as diabetes [7], cancer [8,9,10], and osteoporosis [11,12], which have a remarkable impact on physical and emotional well-being [13,14]. The new WHO 2020 guidelines on physical activity and sedentary behavior address all age groups, with specific recommendations for pregnant and postpartum women and people living with chronic conditions or disability [15]. WHO also amplifies the urgent need to increase priority and investment directed towards services to promote and set PA interventions for the population at large [15].

In the past decade, there has been a marked increase in the number of PA interventions at the community level aiming to address the global public health problem of insufficient PA. Although systematic reviews and meta-analyses support the effectiveness of community-based PA interventions with positive outcomes [16,17,18], it is difficult to identify the most critical components regarding characteristics and type of exercise sessions, duration of intervention, mode of delivery, development of participatory processes, target population subgroups, or regarding strategies for successful implementation in real-world settings [19].

In the field of cardiovascular diseases (CVD), community-based PA programs have been shown not only to determine a positive impact on hypertension, diabetes, obesity/overweight, and altered lipid profile in people with cardiovascular risk factors, but also on improving sustained PA levels [20,21,22,23,24]. In a previous study, our group reported on the feasibility of implementing a physical exercise program (“ACTIVA Murcia Program”) recommended by primary healthcare professionals for primary prevention of ischemic cardiovascular disease [25]. Over 5 years, 3,656 cardiovascular risk patients participated in the program, and after 10 weeks, beneficial effects on physical fitness and decreases in body weight and body mass index (BMI) were recorded. In addition, HRQL was also significantly better in all domains of the Short Form Health Survey (SF-36) after completion of the program as compared with the baseline both in men and women [25]. In a recent study, two modifications of the community-based PA “ACTIVA Murcia Program”, based on differences of training progression and duration of the exercise program of 3 months versus 6 months, were compared to assess adherence and changes of lipid profile, body composition, and blood pressure in older people with cardiovascular risk factors [26]. The results showed that participants involved in the longer program with individualized progression of the loads had greater adherence to physical exercises and a decrease in diastolic blood pressure (DBP). In this study, however, differences between the two types of exercise programs regarding HRQL and changes of physical condition were not evaluated.

Therefore, the present study was conducted to determine whether the characteristics of these two community-based exercise programs for subjects with cardiovascular risk factors were associated with differences in their effects on HRQL and improvement of the participants’ physical condition.

## 2. Materials and Methods

### 2.1. Study Design and Participants

This was a prospective observational cohort study, which was carried out in the Region of Murcia (Spain) and consisted of comparing the effects of two community-based exercise programs on exercise adherence, cardiometabolic biomarkers, and body composition in subjects with cardiovascular risk factors. Detailed description of the characteristics and findings of this study have been previously reported [26]. The present analysis focused on the evaluation of different parameters in the same study population, including the impact of these two community-based exercise programs on the participants’ HRQL and physical condition.

Briefly, participants were subjects of both sexes, aged 30–65 years, attended in the primary healthcare centers of four cities in the Region of Murcia, in whom some of the following cardiovascular risk factors were present, including: sedentary behavior, active smoking, hypertension, prediabetes, overweight or obesity, and alterations of the lipid profile. Subjects diagnosed with cardiovascular disease (e.g., coronary heart disease, is-chemic stroke), blood pressure 180/110 mmHg or higher, uncontrolled metabolic disease, or musculoskeletal diseases limiting or worsening with exercise were excluded.

**Figure 1:**
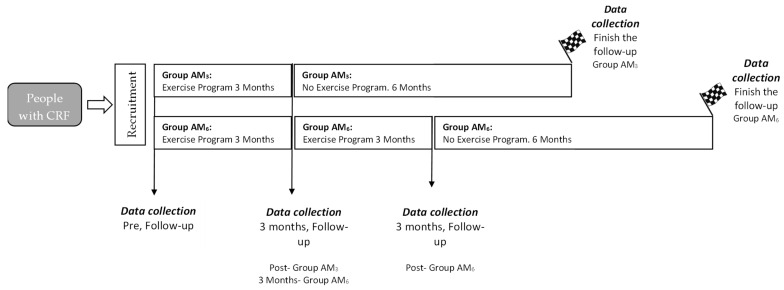
Diagram of the implementation of the AM_3_ and AM_6_ physical activity programs (CRF: cardiovascular risk factors).

Eligible subjects were offered free involvement in a PA program supported by the government of the Region of Murcia and implemented in sports facilities close to the patient’s home. Essential features were that PA was indicated by primary care professionals and exercises were supervised by instructors who were graduates in Sport Sciences. The two programs, the AM_3_ and AM_6,_ run in parallel in two sports centers, one developing the AM_3_ program and the other, the AM_6_ program. Recruitment of subjects started in October 2017 and those who agreed to participate were referred to the sports center closest to their home. The study finished in December 2017 (for the AM_3_) program and in March 2018 (for the AM_6_ program). In all cases, a 6-month follow-up period after completion of the program was established (Figure 1).

It was hypothesized that a physical activity (PA) training program of a longer duration (6 months) combined with an individualized training load (AM_6_) would result in a more favorable impact on HRQL and physical condition than a shorter (3 months) and non-individualized PA training program (AM_3_) both at the end of the programs and after a 6-month follow-up period. A greater effect of the AM_6_ program on these variables (HRQL and physical condition) would further strengthen the previously reported greater benefits of the AM_6_ as compared with the AM_3_ on adherence, cardiometabolic markers, and body composition [26]. Therefore, confirmation of the hypothesis would support the general recommendation of this particular AM_6_ program rather than the AM_3_ to older subjects with cardiovascular risk factors considered eligible for PA prescribed by their primary care professionals.

The study was approved by the Institutional Review Board (code CE101702) and signed informed consent was obtained from all participants.

### 2.2. Community-Based Exercise Programs

Eligible participants were users of the public healthcare system who agreed to perform PA as part of the management of their cardiovascular risk factors. They aimed to make a change in their lifestyle habits and to reduce the risk of overt cardiovascular disease. The PA programs were different in regard to both the duration and type of training. The AM_3_ had a duration of 3 months; the AM_6_, a duration of 6 months (AM is the acronym of ACTIVA Murcia). Both consisted of uninterrupted exercise sessions, followed by a 6-month follow-up without PA.

All PA programs were conducted and supervised by professional sport monitors. The AM_3_ included three 1 h exercise sessions per week (a total of 30 sessions), with a resting period between sessions of 24–48 h. Sessions included a 5 min warm up, 25 min of aerobic circuit and strength exercises, and 5 min of static exercises. The training load was progressive and not individualized. The AM_6_ program also included three 1 h exercise sessions per week (total 72 sessions) with a rest period of 24–48 h. However, the intensity of training load was individualized according to the participant’s level of physical fitness. The general scheme of warm up, strength circuit or aerobic exercises, and cool down phases was similar to the AM_3_. A full description of the exercises, training mode, rates of perceived exertion, and other details of the AM_3_ and AM_6_ programs have been published previously [26]. Subjects had to have an attendance of greater than 65% of the sessions to finish the program.

### 2.3. Outcome Measures

Outcome measures were HRQL and physical condition in participants in the two community-based AM_3_ and AM_6_ programs. Data were collected before starting and after finishing the 3-month or the 6-month PA programs, and after a follow-up observation period of 6 months.

### 2.4. Health-Related Quality of Life (HRQL)

HRQL was evaluated using a Spanish-validated version of the Short Form 36 Health Survey (SF-36) [27]. The SF-36 measures eight multi-item dimensions of health: physical functioning (10 items), social functioning (2 items), role limitations due to physical health (4 items), role limitations due to emotional problems (3 items), perceived mental health (5 items), energy/vitality (4 items), bodily pain (2 items), and general health perception (5 items). For each dimension, item scores are coded, summed, and transformed onto a scale from 0 to 100, with higher scores representing better health status. A remaining single item provides an indication of perceived change in general health status over the last year (health transition).

### 2.5. Physical Condition

The maximum aerobic capacity or maximal oxygen consumption (VO_2_ max, mL/kg/min) was measured using the 1-mile Rockport Walk Test [28], in which the participant should walk the full mile (1609 m) without jogging after a warm up for 5–10 min. Body strength was evaluated through a medicine ball explosive power test, which involves throwing the ball vigorously as far as possible, starting with the ball held with the hands and brought back behind the head [29]. Flexibility was evaluated using the traditional seat and reach test [30], sitting on the floor with legs stretched out straight ahead, and score recorded to the nearest centimeter as the distance reached by the hand (one on top of the other, facing palms down), holding the position for approximately 2 s. Postural stability (balance) was measured with the flamingo balance test, standing on one leg and scoring the number of mistakes made within one minute [31]. In all physical condition tests, three attempts were made. Measurements were made before and after the end of the MA_3_ and MA_6_ programs. Participants in the MA_6_ program underwent an intermediate testing coinciding with the end of the MA_3_ program.

### 2.6. Satisfaction with the Program

At the end of the program, participants completed a seven-item questionnaire regarding improvement in physical fitness and mood (categorized as “a little”, “somewhat better”, and “much better”), and willingness to continue exercising regularly (categorized as “probably no”, “probably yes”, and “yes, for sure”).

### 2.7. Statistical Analysis

The analysis was based on the per-protocol (PP) data set corresponding to those participants who finished the study. Categorical variables are expressed as frequencies and percentages and continuous variables as mean and standard deviation (±SD). For the comparison between groups of the initial characteristics, the Student’s t test was used for quantitative variables (age, weight, and BMI) and the chi-square test or the Fisher’s exact test for categorical variables (sex and cardiovascular risk factors). The analysis of covariance (ANCOVA) was used for the comparison of quantitative variables between the study groups: dependent variable (HRQL and physical condition at 3 months, final, or 6 months follow-up); fixer factors (AM_3_ and AM_6_ program and sex); and covariates (baseline values and age). Within-group comparisons were analyzed with the analysis of variance (ANOVA) for repeated measures with one study factor: time (baseline, 3 months, final and 6 months follow-up); post hoc analyses were performed with the Bonferroni’s correction. The SPSS version 25.0 (IBM Corp., Armonk, NY, USA) was used for data analysis, with a *p* < 0.05 value as statistically significant.

## 3. Results

A total of 108 subjects recruited from primary care centers in four cities of the Region of Murcia (Spain) by their physicians were assessed for eligibility, but 15 were excluded (not meeting the inclusion criteria (10), decline to participate (5)). Of the remaining 93 recruited subjects, 51 were included in the AM_3_ exercise program and 42 in the AM_6_ exercise program. The mean of the study population was 59.3 ± 8.4 years. Women accounted for 77.4% of participants. Dyslipidemia (77.3%), high blood pressure (64.7%), obesity (51.7%), overweight (48.3%), and prediabetes (37.4%) were the most common cardiovascular risk factors. Baseline data are shown in Table 1.

### 3.1. Changes in HRQL

Results obtained in the different subscales of the SF-36 questionnaire at baseline, at the end of the PA program, and after 6 months of follow-up in the AM_3_ and AM_6_ groups are shown in Table 2. In general, the AM_6_ program was associated with increases in more dimensions of HRQL than the AM_3_ program.

**Figure 2:**
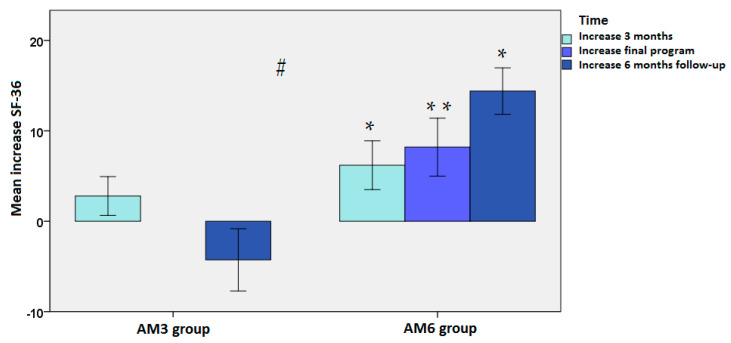
Mean changes in the subscale of physical functioning in the ACTIVA Murcia (AM) exercise programs of 3 months (AM_3_) and 6 months (AM_6_): * *p* < 0.05 and ** *p* < 0.001 as compared with baseline using ANOVA for repeated measures with one study factor (time); post hoc analyses were performed with the Bonferroni’s correction; # *p* < 0.003 at 6 months of follow-up of the AM_3_ vs. AM_6_ using ANCOVA: dependent variable (physical functioning at 6 months follow-up); fixer factors (program and sex); covariates (baseline values and age).

In the subscale of physical functioning, although participants in the AM_3_ program showed significantly better scores at baseline than participants in the AM_6_ program (79.4 ± 17.6 vs. 66.9 ± 20.6, *p* = 0.021), at 6 months of follow-up after the end of training, participants in the AM_6_ program showed significantly higher scores than those in the AM_3_ program (80.8 ± 17.8 vs. 75.1 ± 21.3, *p* = 0.003). Moreover, within-group differences in the AM_6_ program as compared with baseline were statistically significant at all time points (*p* < 0.05 at 3 months and 6 months of follow-up, and *p* < 0.001 at 6 months). Mean changes in the two study groups are shown in Figure 2.

In the subscale of mental health, scores at 6 months of follow-up were significantly higher in the AM_6_ group than in the AM_3_ group (76.0 ± 17.1 vs. 67.6 ± 16.4, *p* = 0.037). Additionally, within-group differences at 6 months and 6 months of follow-up as compared with baseline were significant (*p* < 0.05). In the dimension of bodily pain, within-group differences in the AM_6_ group at 6 months and at 6 months of follow-up as compared with the baseline were statistically significant (*p* < 0.05).

**Figure 3:**
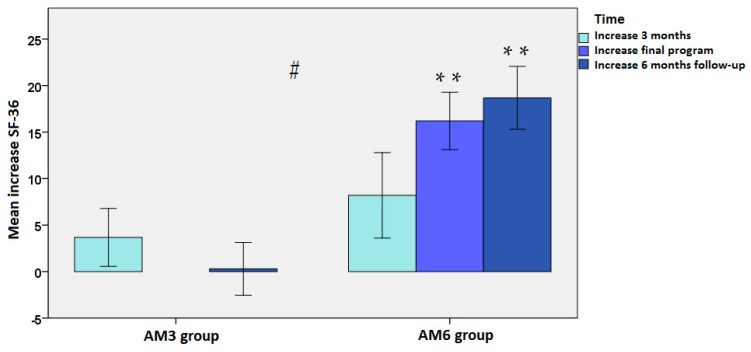
Mean changes in the subscale of energy/vitality in the ACTIVA Murcia (AM) exercise programs of 3 months (AM_3_) and 6 months (AM_6_): ** *p* < 0.001 as compared with the baseline using ANOVA for repeated measures with one study factor (time); post hoc analyses were performed with the Bonferroni’s correction; # *p* = 0.05 at 6 months of follow-up of the AM_3_ vs. AM_6_ using ANCOVA: dependent variable (physical functioning at 6 months follow-up); fixed factors (program and sex); covariates (baseline values and age).

In the subscale of energy/vitality, scores were significantly higher in the AM_6_ group than in the AM_3_ group at 6 months of follow-up (69.5 ± 19.5 vs. 56.3 ± 18.6, *p* = 0.009) and also at the end of each program (*p* = 0.048). Significant within-group differences as compared with the baseline at 6 months and at 6 months of follow-up were found in the AM_6_ program (*p* < 0.001). Mean changes in the two study groups are shown in Figure 3.

General health improved significantly in the AM_6_ group as compared with the AM_3_ group (63.9 ± 18.9 vs. 57.3 ± 15.1, *p* = 0.049). Within-group differences at 6 months of follow-up as compared with baseline were significant in the AM_6_ group (*p* < 0.05).

In the remaining subscales of social functioning, role physical, and role emotional, changes in HRQL were not significant in any of the AM_3_ and AM_6_ groups.

Change in health status over the last year showed a decrease, which was more noticeable in the AM_6_ group than in the AM_3_ group, particularly at 6 months of follow-up (20.8 ± 14.1 vs. 40.4 ± 19.5, *p* = 0.001).

### 3.2. Changes in Physical Condition

Results obtained in the four variables of physical condition in the study groups are shown in Table 3. The maximum aerobic capacity or maximal oxygen consumption (VO_2_ max) increased significantly in both AM programs at the end of 3 and 6 months, respectively, as compared with baseline (*p* < 0.05 for the AM_3_ group, and *p* < 0.001 for the AM_6_ group). At the end of the programs, increases in VO_2_ max were significantly higher in the AM_6_ group (*p* = 0.001).

Strength improved also significantly (*p* < 0.001) in the two groups at the end of the exercise programs when compared with the baseline, but at 3 months (end of the AM_3_ program and intermediate assessment of the AM_6_ program), results were significantly better for the AM_3_ group (*p* = 0.023).

Similar findings were obtained in the improvement of flexibility, with significant differences in both study groups at the end of programs as compared with the baseline (*p* < 0.001). However, final flexibility values were significantly higher in the AM_6_ program (9.57 ± 4.93 cm) than in the AM_3_ program (5.0 ± 6.96 cm) (*p* = 0.046).

In the balance test, significant improvements (*p* < 0.05) at the end of the program as compared with the baseline were found in the two study groups, but differences between the AM_3_ and AM_6_ were not observed.

Subjects in the AM_3_ group attended a mean of 78.6 ± 10.5 sessions; subjects in the AM_6_ group, a mean of 76.0 ± 7.8 (*p* = 0.232).

### 3.3. Participants’ Satisfaction

A total of 37.9% and 31.8% of participants in the AM_3_ and AM_6_ groups reported “much better” physical fitness at the end of the program, with 55.2% and 54.5% reporting “somewhat better”. Similar results were obtained for mood improvement, with 34.5% and 18.2% of participants in the AM_3_ and AM_6_, respectively, reporting “much better” mood, and 62.1% and 68.2% reporting “somewhat better”.

For willingness to continue exercising regularly, more participants in the AM_6_ program than in the AM_3_ reported “yes, for sure” (50% vs. 37.9%). “Probably yes” was selected by 55.2% of participants in the AM_3_ group and 40.9% in the AM_6_ group, and “probably no” by 6.9% and 9.1%, respectively.

## 4. Discussion

Physical inactivity and sedentary behavior are associated with increased risk of CVD incidence and mortality. A recent systematic review and meta-analysis showed dose-dependent associations between sitting time (and television viewing time) with CVD, cancer, and all-cause mortality [32]. In a cohort study of 17,465 United States participants in the prospective REasons for Geographic and Racial Differences in Stroke (REGARDS) study followed over a median of 10.3 years, there was an inverse association between all-cause, all-CVD, and all-cancer mortality and the evolutionary-concordance lifestyle score, which was a composite score that included diet, alcohol intake, PA, sedentary behavior, waist circumference, and social network size [33]. Therefore, leveraging the time inverse relationship between sedentary and active behaviors by replacing sitting with light or moderate-intensity activity can have important health benefits, particularly among subjects at cardiovascular risk.

Reducing sedentary behavior by increasing PA at the community level is an effective intervention aimed at CVD prevention, but still remains a difficult challenge. In our experience, implementation of a preventive PA exercise program recommended in primary care by general practitioners with the regional governmental support has shown health benefits and improvements in physical fitness [25,26]. The present study adds evidence of a further improvement in HRQL. Physical exercise has the capacity to induce positive effects on well-being, enhancing self-efficacy, reducing depressive symptoms, improving mood, and building social support [34]. We found that participants in the MA_6_ program characterized by individualized progressive intensity of training load, with a duration of 6 months, reported better effects on HRQL than those in the MA_3_ program, in which training load was progressive but not individualized and which had a duration of 3 months. Independently of differences in the progression of training (individualized vs. non-individualized), the longer duration of the MA_6_ program favored adherence [26] and participants had a better perception of physical and mood improvement, contributing to promoting a positive change in lifestyle. This was reflected by a higher percentage of participants in the AM_6_ program indicating their firm desire to continue exercising.

Participants in the AM_6_ program as compared with those in the AM_3_ program showed significantly higher scores in the subscales of physical functioning, mental health, energy/vitality, and general health. Differences were particularly remarkable in the subscale of energy/vitality both at the end of the programs and after 6 months of follow-up. Mental health and general health at 6 months of follow-up were also scored significantly higher by participants in the AM_6_ group. On the other hand, significantly better scores at the end of the AM_6_ program and after 6 months of follow-up as compared with baseline were observed in five subscales (physical functioning, mental health, energy/vitality, bodily pain, and general health), whereas there were no significant within-group differences in any subscale in the AM_3_ group. However, despite overall improvements in HRQL associated with the community-based PA programs, the perceived health status over the last year decreased during the study period, being significantly better at 6 months of follow-up in the AM_3_ group. It could be possible that increasing awareness of participants of their abilities and limitations to perform PA elicited by the longer program made them perceive a worse health transition during the last year.

The beneficial effect of PA on HRQL documented in the present study is consistent with results reported by other authors. In two Brazilian populations, 3 months of community-based programs (two to three times/week aerobic exercise, circuit resistance training, and stretching exercises for 1 h each time) were associated with significant improvements in physical functioning, general health, and role physical as compared with controls [35]. Tsai, et al., [36] evaluate the effects of moderate-intensity aerobic exercise group training for 3 sessions/week over 10 weeks on quality of life and blood pressure in patients with mild to moderate hypertension, and showed higher scores on seven out of eight subscales (*p* < 0.05) of the SF-36. In this study, however, patients were recruited from hospital outpatient clinics and the intervention was not delivered in a community-based setting. In the study of Arija, et al., [20], a community-based, randomized, controlled, intervention program among adults attending primary care clinics to promote PA over 9 months significantly improved cardiovascular risk factors, but the impact on HRQL was not evaluated.

Changes in physical condition of participants with cardiovascular risk factors resulting from benefits of PA are well-established [37]. We found improvements in VO_2_ max, strength, flexibility, and balance among participants in both PA programs, although VO_2_ max and flexibility showed higher improvements in the AM_6_ group with significant differences as compared with the AM_3_ group.

Limitations of the study include the relatively small number of participants in the two community-based PA programs, but the comparison of the two exercise programs with different duration and load progression training characteristics can be helpful for optimizing the design of further studies. However, it should be noted that the improvements in HRQL and physical condition found for the AM_6_ program cannot be dissociated as primarily due to the longer duration of PA or due to the individualized characteristics of the training program. The assessment of potential differences between men and women of the impact of community-based PA programs on HRQL may be also evaluated in future studies.

## 5. Conclusions

Subjects with cardiovascular risk factors who participated in a community-based physical exercise program characterized by individualized progressive training loads for 6 months could benefit from a greater improvement in HRQL than those involved in a program of non-individualized training loads for 3 months. The AM_6_ program also elicited better improvements in the physical condition of participants and a greater willingness to continue exercising.

## Figures and Tables

**Table 1:** Baseline characteristics of the study population.

Variables	ACTIVA Murcia Community-Based Exercise Program		*p* Value
	AM_3_ (3 Months) (n = 51)	AM_6_ (Six Months) (n = 42)	
Age, years, mean ± SD	59.2 ± 7.4	59.4 ± 8.9	0.948
Sex, % women	82.4	71.4	0.210
Weight, kg, mean ± SD	78.7 ± 16.0	75.9 ± 13.5	0.477
Body mass index (BMI), kg/m^2^, mean ± SD	30.6 ± 5.3	29.5 ± 4.3	0.659
Cardiovascular risk factors, %			
Hypertension	62.7	66.7	0.694
Dyslipidemia	78.4	76.2	0.797
Obesity, BMI ≥ 30 kg/m2	51.0	52.4	0.893
Overweight, BMI ≥ 25 and < 30 kg/m^2^	49.0	47.6	0.893
Prediabetes	39.2	35.7	0.728
Current smoker	9.8	7.1	0.648

SD. Standard deviation; Student’s *t* test for continuous variables; chi-square or Fisher’s exact test for categorical variables.

**Table 2:** Differences in HRQL between the two community-based exercise programs.

ACTIVA Murcia Programs	Physical Functioning	Social Functioning	Role Physical	Role Emotional	Mental Health	Energy/ Vitality	Bodily Pain	General Health	Health Transition
AM_3_ (n = 51)									
Baseline	79.4 ± 17.6	79.4 ± 19.7	83.1 ± 34.7	78.4 ± 40.1	69.3 ± 17.4	56.0 ± 22.6	68.8 ± 24.9	54.9 ± 17.4	44.8 ± 18.2
3 months	82.2 ± 18.0	84.2 ± 19.3	78.7 ± 37.5	85.3 ± 34.0	71.8 ± 14.3	59.7 ± 16.5	71.6 ± 22.9	60.0 ± 16.3	28.7 ± 19.6 *
6 months follow-up	75.1 ± 21.3	79.4 ± 18.7	77.9 ± 37.3	67.6 ± 43.0	67.6 ± 16.4	56.3 ± 18.6	70.2 ± 22.7	57.3 ± 15.1	40.4 ± 19.5
AM_6_ (n = 42)									
Baseline	66.9 ± 20.6	80.7 ± 18.8	64.5 ± 39.6	56.9 ± 48.6	65.5 ± 25.2	50.4 ± 25.0	60.7 ± 26.5	54.4 ± 20.6	46.9 ± 27.9
3 months	73.9 ± 17.9 *	82.3 ± 18.0	70.8 ± 38.1	69.4 ± 43.9	69.2 ± 23.8	59.2 ± 22.6	69.5 ± 21.9	55.3 ± 16.9	38.5 ± 20.8
6 months	77.3 ± 14.8 *	84.9 ± 16.5	71.9 ± 38.5	70.8 ± 42.1	74.5 ± 21.6 *	67.3 ± 20.3 *	75.2 ± 21.3 *	60.0 ± 20.3	28.1 ± 22.5 *
6 months follow-up	80.8 ± 17.8 *	82.8 ± 22.4	81.2 ± 36.3	79.2 ± 36.5	76.0 ± 17.1 *	69.5 ± 19.5 *	74.0 ± 26.9 *	63.9 ± 18.9 *	20.8 ± 14.1 *
AM_3_ vs. AM_6_									
Baseline	*p* = 0.021	*p* = 0.824	*p* = 0.076	*p* = 0.10	*p* = 0.556	*p* = 0.565	*p* = 0.334	*p* = 0.956	*p* = 0.756
3 months	*p* = 0.712	*p* = 0.892	*p* = 0.987	*p* = 0.233	*p* = 0.872	*p* = 0.887	*p* = 0.899	*p* = 0.589	*p* = 0.08
6 months follow-up	*p* = 0.003	*p* = 0.534	*p* = 0.256	*p* = 0.211	*p* = 0.037	*p* = 0.009	*p* = 0.121	*p* = 0.049	*p* = 0.001
Final AM_3_ vs. AM_6_	*p* = 0.081	*p* = 0.823	*p* = 0.898	*p* = 0.342	*p* = 0.675	*p* = 0.048	*p* = 0.433	*p* = 0.709	*p* = 0.821

AM_3_: ACTIVA Murcia exercise program of 3 months; AM_6_: ACTIVA Murcia exercise program of 6 months; baseline: statistical significance when comparing baseline values with the Student’s *t* test; 3 months, 6 months follow-up and final AM_3_ vs. AM_6_: statistical significance when performing ANCOVA for the comparison of HRQL of life at 3 months, final or 6 months follow-up between the study groups; baseline values and age were considered as co-variables in the analysis; * within-group statistically significant differences as compared with baseline values. ANOVA for repeated measures with one study factor (time); post hoc analyses were performed with the Bonferroni’s correction.

**Table 3:** Differences in physical condition at different time points between the two community-based exercise programs.

ACTIVA Murcia Programs	VO_2_ Max mL/kg/min	Body Strength m	Flexibility cm	Balance Number of Mistakes
AM_3_ (n = 51)				
Baseline	23.7 ± 9.4	4.55 ± 0.10	0.17 ± 7.48	2.51 ± 3.85
3 months	27.7 ± 9.0 *	5.07 ± 0.93 *	5.00 ± 6.96 *	1.18 ± 2.43 *
AM_6_ (n = 42)				
Baseline	28.6 ± 10.9	4.89 ± 1.16	3.53 ± 5.92	2.62 ± 3.09
3 months	30.9 ± 9.8	5.00 ± 1.23	6.78 ± 4.73	2.03 ± 2.39
6 months	41.1 ± 11.4 *	5.18 ± 1.03 *	9.57 ± 4.93 *	0.85 ± 1.35 *
AM_3_ vs. AM_6_				
Baseline	*p* = 0.081	*p* = 0.213	*p* = 0.067	*p* = 0.938
3 months	*p* = 0.879	*p* = 0.023	*p* = 0.689	*p* = 0.126
Final AM_3_ vs. AM_6_	*p* = 0.001	*p* = 0.078	*p* = 0.046	*p* = 0.412

AM_3_: ACTIVA Murcia exercise program of 3 months; AM_6_: ACTIVA Murcia exercise program of 6 months; baseline: statistical significance when comparing baseline values with the Student’s *t* test; 3 months and final AM_3_ vs. AM_6_: statistical significance when performing ANCOVA for the comparison of physical condition at 3 months or final between the study groups; baseline values and age were considered as co-variables in the analysis; * within-group statistically significant differences as compared with baseline values. ANOVA for repeated measures with one study factor (time); post hoc analyses were performed with the Bonferroni’s correction.

## Data Availability

Study data are available from the authors upon request.
